# A New Febrifuge

**Published:** 1849-01

**Authors:** 


*A New Febrifuge.*—The Journal de Pharmacie and de Chimie contains an article of M. Duchassaing on the bark of the baobab, or Adansonia digitata, which he pronounces to be a succedaneum of Peruvian bark. Golbery and Frank mention that the Indians of Senegal make an agreeable beverage with the pulp which surrounds the seeds of the fruit, and that this drink is employed by them in the fevers of the country. Adanson, a French botanist, who first introduced the plant, states, in an account to the Academy in 1761, that he kept himself free from the epidemic fevers of Senegal by using a decoction of the dried leaves of the plant. Dr. Duchassaing now announces that a decoction of the bark of the baobab (which grows plentifully in Senegal, and along the whole of northern Africa as far as Abyssinia) is all-powerful in ague. He takes two pounds and five drachms of water to one ounce of the bark; the decoction is very apt to spoil, but this defect is remedied by a few drops of sulphuric acid or alcohol. The author has used the bark extensively, and found it very efficacious even in those cases where quinine had failed once. Planters of Guadaloupe, to whom Dr. Dachassaing communicated the virtues of this plant, use it now exclusively for their negroes, and have never recourse to quinine (which is very expensive in that colony) except in peculiar cases. It is to be doubted whether the baobab will ever supersede quinine; but if further experiments with it are successful, it might be employed where quinine is found too dear, and as possessing, for many cases, a sufficient amount of activity; in fact, it may become, if not an *alter ego,* at least a succedaneum to quinine.—*London Lancet.*

